# The Complex of Copper (II) and Zoledronic Acid: Relevance to Oxidative Death of Leukemia Cells in the Bone Marrow

**DOI:** 10.3390/ijms27062800

**Published:** 2026-03-19

**Authors:** Elena S. Barskaya, Artemii M. Savin, Kirill V. Chernov, Albina S. Petrova, Maksim S. Abramovich, Yulia A. Maksimova, Alexander S. Dubenskiy, Sergey A. Tsymbal, Anna V. Lantsova, Anna A. Moiseeva, Maria A. Beloglazkina, Roman S. Borisov, Elena K. Beloglazkina, Alexander A. Shtil

**Affiliations:** 1 Department of Chemistry, Moscow State University, 1/3 Leninskie Gory, Moscow 119991, Russia; elenabarskaia44@gmail.com (E.S.B.); mabramovich98@gmail.com (M.S.A.); moiseeva1955@mail.ru (A.A.M.); beloglazkinamaria@mail.ru (M.A.B.); beloglazki@mail.ru (E.K.B.); 2 Center for Molecular and Biological Technologies, ITMO University, 9 Lomonosov Street, Saint-Petersburg 197101, Russia; savin@itmo.ru (A.M.S.); chernov@itmo.ru (K.V.C.); zimbal@itmo.ru (S.A.T.); 3 Institute of Medicine, RUDN University, 6 Miklukho-Maklaya Street, Moscow 117198, Russia; albina.s.petrova@yandex.ru; 4 Department of Physics, Mathematics and Natural Sciences, RUDN University, 6 Miklukho-Maklaya Street, Moscow 117198, Russia; 5 Laboratory of Chemical Analytical Research, Geological Institute of Russian Academy of Sciences, 7 Pyzhevsky Lane Bld. 1, Moscow 119017, Russia; yu.a.maksimova@gmail.com (Y.A.M.); alexchem206@gmail.com (A.S.D.); 6 Laboratory of Bio- and Cheminformatics, School of Computer Science, Physics and Technology, Higher School of Economics, 6 25th Line of Vasilievsky Island, Saint-Petersburg 199106, Russia; 7 Institute of Experimental Oncology and Carcinogenesis, Blokhin National Research Center of Oncology, 24 Kashirskoye Shosse, Moscow 115522, Russia; lantsova1979@mail.ru; 8 A.V. Topchiev Institute of Petrochemical Synthesis, Russian Academy of Sciences, 29 Leninsky Avenue, Moscow 119071, Russia; borisov@ips.ac.ru; 9 Institute of Cyber Intelligence, National Research Nuclear University MEPhI, 31 Kashirskoye Shosse, Moscow 115409, Russia

**Keywords:** copper, redox reactions, electrochemistry, zoledronic acid, cell death, medicinal chemistry

## Abstract

Copper–organic compounds are being investigated as antitumor candidates. Besides their efficacy as cytotoxic agents alone, the oxidative potential of electrochemical Cu^2+^-to-Cu^1+^ transition emerges as an attractive approach for elimination of tumor cells otherwise resistant to chemotherapy. To minimize side effects of the potent oxidative burst upon Cu(II) reduction, the metal cations should be delivered to the tumor site. Taking advantage of the ability of bisphosphonates to accumulate in the bone, we synthesized a Cu(II) complex of zoledronic acid (ZA), an FDA-approved drug for prevention of bone destruction. The **CuZA** complex obtained upon precipitation of ZA and different copper salts (sulfate, chloride or perchlorate) were structurally identical, consisting of two organic moieties coordinated by three metal cations. Combined treatment with water-soluble formulations of **CuZA** and cysteine triggered rapid death in human cell lines. This effect was achievable with non-toxic concentrations of **CuZA** and cysteine alone. Importantly, the K562 chronic myelogenous leukemia cells that demonstrated an attenuated response to the 3d generation Bcr-Abl tyrosine kinase inhibitor in the medium conditioned by bone marrow-derived fibroblasts, were readily killed by **CuZA**–cysteine combination. Thus, oxidative burst upon metal reduction in **CuZA** complexes emerges as a promising method of eradication of tumor cells in the bone microenvironment.

## 1. Introduction

The osseous tissue, and the bone marrow in particular, is a frequent site of cancer metastasis. Bone colonization is a negative prognostic factor that indicates the disease progression. Tumor cells that reside in the bone marrow are protected from chemo- and radiotherapies due to numerous epigenetic mechanisms [1]. Cell–matrix interactions via integrin receptors, as well as soluble compounds excreted by the stroma and infiltrating blood cells, all contribute to the formation of the survival niche for tumor cells (ref. [2] and refs. therein). This microenvironment promotes the reprogramming of transcriptional and metabolic profiles of tumor cells, thereby altering their responses to otherwise cytotoxic stimuli. Tissue damage by tumor-derived proteolytic enzymes leads to pathological fractures and the pain syndrome, further burdening the patient’s state.

Several aspects of tumor biology are relevant to the therapy of bone metastases. First, the resident tumor cells represent a population that survived after the repetitive rounds of treatment. Therefore, the blocked apoptotic pathways provide survival advantage. Consequently, involvement of non-apoptotic modes of cell death emerges as a mechanistically substantiated approach. Next, selective elimination of tumor cells for the sake of sparing non-malignant counterparts is unlikely to be straightforward. In contrary, the therapeutic strategies should presume the eradication of the pro-oncogenic microenvironment, that is, the bone marrow purge. These considerations argue against single target therapies to combat resident tumor cells in the bone. Indeed, clinical efficacy of Bcr-Abl tyrosine kinase inhibitors (TKI) against chronic myelogenous leukemia (CML) cells in the bloodstream is higher than in the bone marrow [3,4,5].

Instead, the necrosis-like cues deserve to be regarded as an alternative. The primary loss of the plasma membrane integrity is a major factor of induction of cell death. Among a variety of stimuli, reactive oxygen species (ROS) are the established metabolites capable of triggering the irreparable damage of the plasma membrane and the membrane organelles followed by cell death. Most importantly, the ROS-sensitive death pathways remain functional in cells that acquired drug resistance [6,7]. Apparently, ROS generation in antitumor therapy is a double-edge sword since the protection of normal tissue elements is problematic. Therefore, oxygen burst should be localized to the disease site. In this scenario the potency of ROS against tumor cells and their microenvironment is expected to be significant whereas the systemic toxicity is minimized.

Recently we reported a remarkable cytocidal activity of ROS generated upon the electrochemical reduction of Cu(II) to Cu(I). This effect was achievable by combining CuO nanoparticles or copper–organic complexes with N-acetylcysteine (NAC) or physiological reducing agents (i.e., cysteine or ascorbate) [8]. Importantly, the Cu^2+^-containing agent and the reducing amino acids were at virtually non-toxic concentrations whereas potentiation of the cytotoxic efficacy of the combination reached 2–3 orders of magnitude. One may hypothesize that the delivery of Cu^2+^ to the bone and subsequent administration of the reducing agent would locally trigger lethal ROS burst.

In the present study we approached this assumption by the design and synthesis of Cu(II) complex with zoledronic acid (ZA), an FDA-approved bisphosphonate [9,10,11]. This drug demonstrated a good clinical efficacy in preventing metastatic bone resorption in patients with breast, renal and prostate cancer, as well as myeloma [12,13,14,15]. Most importantly, the new water-soluble formulations of the complex (termed **CuZA**) in combinations with cysteine readily cultured CML cells otherwise protected from the Bcr-Abl inhibitor in the medium conditioned by bone marrow-derived fibroblasts.

## 2. Results

### 2.1. Chemistry

#### 2.1.1. Synthesis and Characterization of **CuZA**

**Figure 1:**
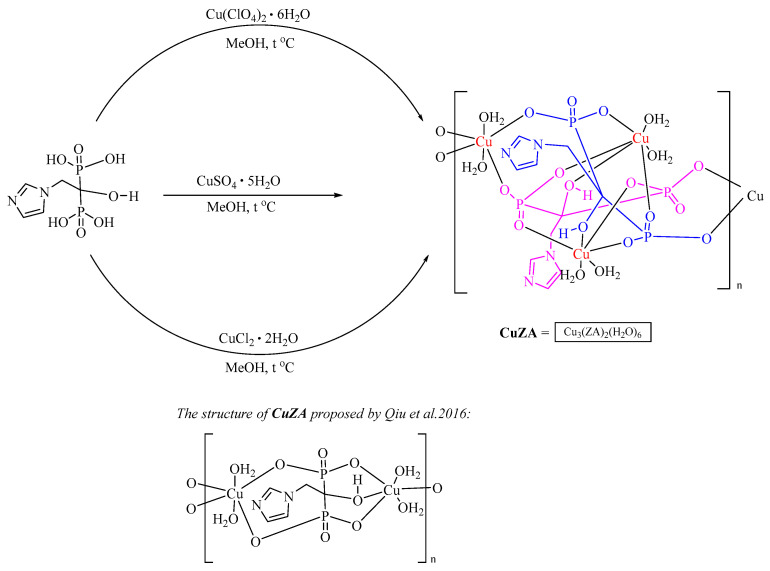
Synthesis of **CuZA** complex. Copper atoms are colored in red, two ZA molecules are shown in blue and violet. Note that the perchlorate, sulfate or chloride salts yielded the same **CuZA** complex [16].

The initial copper (II)–ZA complex was prepared according to the previously described method starting from CuSO_4_ [16]. Importantly, we realized that the structural formula of the complex based on X-ray data (Figure 1) needs to be refined. Extending the analysis of the product generated in [16], we employed elemental CHN analysis (Section 4.2), as well as IR and UV–vis spectroscopy (Figures S1 and S2, Supplementary Information). Furthermore, the ICP-MS method was used to determine the amount of copper in the complex. The presence of all equivalent copper atoms in the complex was confirmed by electrochemical data (see Section 2.1.3).

The IR spectra of copper phosphates are typically characterized by intense absorption bands due to vibrations of phosphate anions. Main peaks are observed in the regions of 900–1200 cm^−1^ (P-O stretching vibrations) and 500–650 cm^−1^ (O-P-O deformation vibrations), as well as in the region of 1600–3500 in the presence of hydrated water. The presence of crystal hydrates adds broad bands of OH groups in the region of 3000–3500 cm^−1^ and deformation vibrations of water at 1600–1650 cm^−1^ [17,18,19]. The main characteristic signals in the IR spectrum of **CuZA** are a set of intense bands within the 540–1180 cm^−1^ range, corresponding to vibrations of the PO_4_ fragment, as well as a very broad absorption band in the 2600–3500 cm^−1^ region and a less intense one at 1620–1650 cm^−1^, confirming the presence of a large number of water molecules in the complex (Figure S1, Supplementary Information). Thus, the IR spectroscopy data are consistent with the proposed refinement of **CuZA** structure.

The electronic spectrum of **CuZA** (Figure S2, Supplementary Information) contains an absorption band in the UV region with a maximum at ~330 nm, in the absence of absorption bands in the visible region, which is consistent with the octahedral coordination environment of copper [20] and with the newly suggested formula.

**Figure 2:**
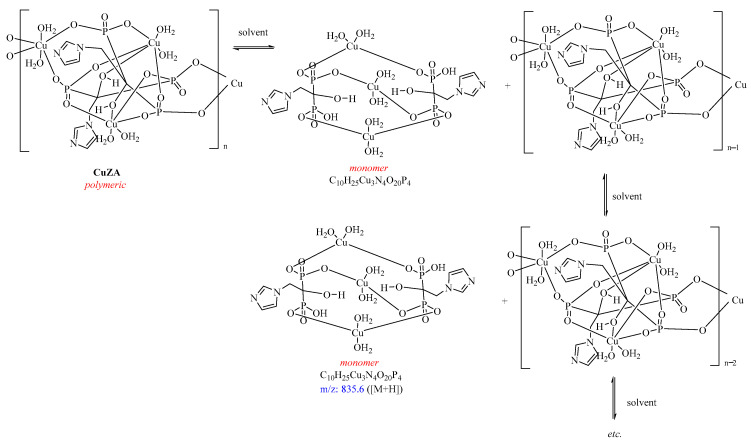
Equilibrium between polymeric and monomeric forms of the **CuZA** complex. See text for details.

To further confirm the structure of **CuZA**, we used MALDI and electrospray ionization (ESI) mass spectrometry. In the electrospray ionization mass spectra (Figure S3, Supplementary Information), a peak of an ion corresponding to the protonated monomer of **CuZA** (molecular formula C_10_H_25_Cu_3_N_4_O_20_P_4_ ([M+H]^+^, *m*/*z* 835.6)) was observed. Additional peaks with the masses [M+(58·n)+H]+ (n – 1–7) and a similar isotope distribution were also registered in ESI spectra, apparently corresponding to sequential coordination by **CuZA** with six acetamide molecules formed in the ESI experiment (solvent DMSO/CH_3_CN, HCOOH) from acetonitrile and water of **CuZA** crystallohydrate (Figure S4). This suggests that the **CuZA** complex exists as equilibrium between the monomeric and polymeric forms (Figure 2). Such a structure has been reported for polymeric supramolecular metal complexes capable of partial dissociation and the release of monomeric fragments into the solution, although to a limited extent [21,22].

Of note, an artifactual (in-source) polymerization may be observed during electrospray ionization: the concentration of the analyte in charged droplets increases sharply due to solvent evaporation. This can lead to the formation of non-covalent aggregates (clusters) or even initiate the reactions between the analyte molecules directly in the ion source, thereby distorting the spectrum. Therefore, we studied the **CuZA** compound by MALDI using AT, CHCA, DCTB and rubrene matrices. No peak with the expected mass was observed in the spectra. However, on CHCA and rubrene, the peaks with a significantly lower *m*/*z* than the monomeric complex (*m*/*z* ≤ 566) were detectable, corresponding either to matrix ions or to decay products of the target compound (Figures S5–S8).

Finally, to unambiguously confirm the identity of the complex synthesized as described in [16], we studied **CuZA** by powder X-ray diffraction and compared the crystal cell parameters with data in the original report (CCDC 901874). The coincidence of these parameters demonstrated the identical structure of both complexes. Finally, comparison of the powder X-ray diffraction data with the parameters of the ZA crystal cell mono- and trihydrate, CCDC 1562048, 1562049 and 693503, respectively) showed no noticeable impurities in the samples.

Our synthetic procedures using copper salts other than sulfate, such as chloride or perchlorate, resulted in the formation of precipitates similar in composition to the complex obtained from CuSO_4_. Thus, the composition of **CuZA** complexes was independent of the metal cation (counter-ion) in the salt. In fact, the same complex was produced from three different salts. The corrected structure is shown in Figure 1.

#### 2.1.2. Reducing Agents

Previously we have demonstrated that NAC was capable of reducing copper(II) in the context of oxide or metal–organic compounds as determined by electrochemical methods and cytotoxicity tests [8,23]. In the present study, we turned to cysteine as a physiological reducing agent in cell culture experiments, whereas its derivative NAC was used in electrochemistry measurements for compatibility with organic solvents.

#### 2.1.3. Electrochemical Assays

We aimed to use cyclic voltammetry (CV) and rotating disk electrode (RDE) voltammetry techniques in DMSO with 0.1 M Bu_4_NClO_4_ as a supporting electrolyte. CV determines the reduction potentials while RDE allows for estimation of the oxidation state of copper [24] and E_1/2_ of redox transition. Both free ZA and **CuZA** complex were extremely poorly soluble in DMSO, as well as in other tested solvents suitable for electrochemical studies such as CH_3_CN or dimethyl formamide. Therefore, CV measurements for these compounds were impossible.

**Figure 3:**
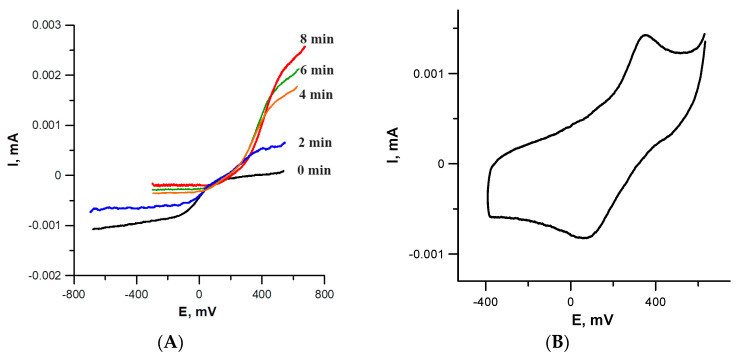
NAC-induced metal reduction in **CuZA**. (**A**) RDE curves of **CuZA** solution upon NAC addition (3 equiv). (**B**) CV of **CuZA** (1 equiv.) + NAC (3 equiv.) solution after complete metal reduction (10 min after mixing).

However, a more sensitive RDE voltammetry allowed to determine the potential of Cu^2+^ → Cu^1+^ redox transition in the complex. NAC was added to **CuZA** solution in an amount sufficient to reduce all copper cations in the complex (3 equiv., one per each Cu cation). Although the initial complex was poorly soluble in DMSO, its solubility increased significantly upon the addition of NAC: note an increased current on the RDE curve (Figure 3A; compare black and colored lines). According to RDE data, Cu^2+^ rapidly disappeared, transforming the complex into a Cu^1+^-containing form. This is demonstrated by gradual change in the current from cathodic (corresponds to reduction) to anodic (corresponds to oxidation) during Cu^1+^ ←→ Cu^2+^ redox transition. Eight min after the addition of NAC, the reduction was complete and the system stabilized; only Cu^1+^ cations were present in the solution. Single Cu^2+^ → Cu^1+^ transition in RDE confirmed the identical coordination environment of all three metal cations in the complex (no interaction between metal cations); therefore, their reduction occurred at the same potential.

For the resulting Cu^1+^ZA complex, due to its higher solubility compared to the starting **CuZA**, a CV curve can be recorded (Figure 3B). The redox potential of Cu^1+^ ←→ Cu^2+^ transition was ~+0.38 V. If a 10-fold excess of NAC was added to **CuZA** solution instead of 3 equiv., the Cu^2+^ → Cu^1+^ reduction occurred instantly. Within 1 min after admixing, only Cu^1+^ remained in the solution according to RDE. Thus, the electrochemical data confirmed rapid Cu^2+^ → Cu^1+^ transition in **CuZA** complex in the presence of the reducing agent.

#### 2.1.4. Water-Soluble Formulations

The **CuZA** complex was practically insoluble in water, soluble in chloroform, very slightly soluble in 95% ethanol, acetone and benzene, and readily soluble in DMSO and dimethyl formamide. We applied an approach [25,26] based on varying pH to obtain a water-soluble salt and its subsequent stabilization with the co-solvent, polyvinylpyrrolidone (Kollidon 17PF). The water-soluble, biocompatible and storage-stable compositions were characterized by mass ratio of **CuZA** complex/0.1 N NaOH/95% ethanol/Kollidon 17PF: 1.2/1/44/22. The sequence of steps and conditions of dissolution were important for the formation of stable solutions of the required concentration. Of note, **CuZA** and Kollidon 17PF must first be co-dissolved in 95% ethanol with stirring at 400 rpm, 60 °C. Then, the resulting concentrate was diluted with 0.1 N NaOH. For cell culture studies, we prepared 10 mM stock solution of **CuZA** (aqueous formulation).

### 2.2. Biological Testing

#### 2.2.1. Reductive Cytotoxicity of CuZA Complexes

**Figure 4:**
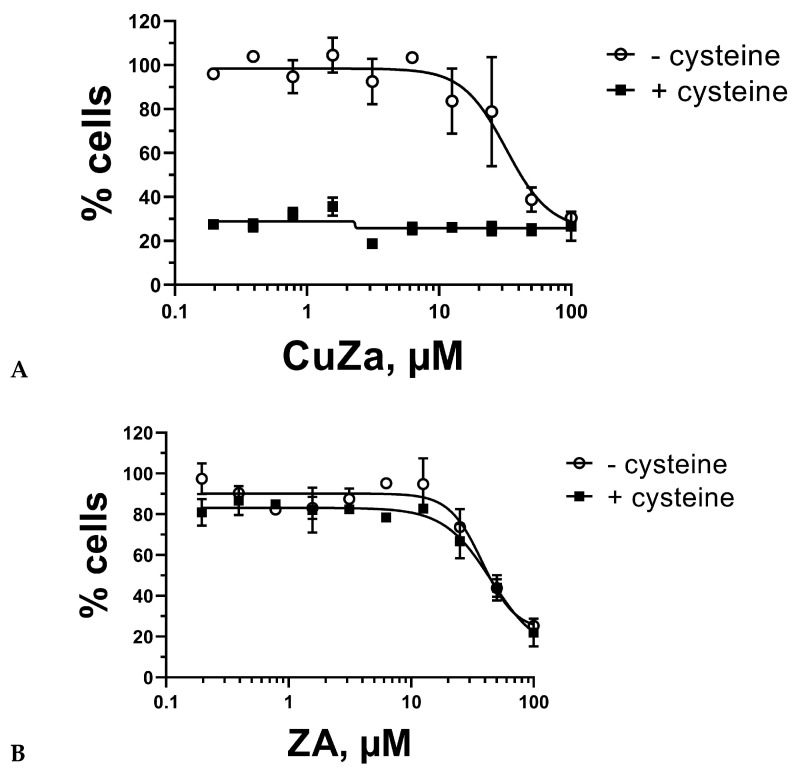
Copper cations are necessary for reductive cytotoxicity. The HS5 fibroblasts were treated with indicated concentrations of **CuZA** ((**A**); top panel) or ZA ((**B**); bottom panel) in the absence or presence of 0.5 mM cysteine for 48 h followed by MTT tests [8]. Values are mean ± standard errors (n = 3 biological replicates, each concentration tested in duplicate).

We tested a broad range of **CuZA** concentrations to determine the doses (0.1–10 µM) that evoked little-to-no cytotoxicity in HS5 bone marrow-derived fibroblasts for at least 48 h of exposure. Cysteine alone was non-toxic at 0.5–1 mM. These results were similar to those determined by us with Cu(II)–organic compounds and NAC for other cell lines [8,23]. In striking contrast, the combinations of as low as 0.2 µM **CuZA** and 0.5 mM cysteine caused a significant cytotoxicity in HS5 fibroblasts: the percentages of MTT conversion, a measure of cell viability, were at the assay’s background (Figure 4A). In contrast, the metal-free ZA alone (up 10 µM) was inert; the addition of 0.5 mM cysteine was without the effect (Figure 4B), indicating that cell death is mechanistically linked to Cu^2+^ reduction. The initial signs of cell death were detectable within 6 h of exposure to the combinations, similarly to CuO or Cu(II) organic compounds in combination with NAC [8]. Most importantly, cell death by the combination of **CuZA** and cysteine was achieved with concentrations that evoked no discernible cytotoxicity if each component was administered alone.

#### 2.2.2. Intracellular vs. Extracellular Distribution of CuZA

We were interested whether **CuZA** complexes accumulate in cultured cells. This parameter was determined based on measurements of inorganic copper in cell lysates (intracellular content) and in culture medium (extracellular content) after incubation with the non-toxic concentration of **CuZA**. Table 1 shows time dependent uptake of **CuZA** by cells of different tissue origin. Intracellular copper content increased over time, with maximum registered by 24 h. Interestingly, cells incorporated only a minor portion of **CuZA**: the metal was detected predominantly in the extracellular medium. These ratios were consistent for adherent (HS5) as well as for suspension (K562) cells (Table 1) indicating a limited accumulation of **CuZA** complexes inside the cells.

#### 2.2.3. Cytotoxic Potency of CuZA–Cysteine Combination

Keeping in mind relatively low intracellular accumulation of **CuZA**, one may argue that reduction of extracellular, not as much intracellular, copper confers ROS-mediated cytotoxicity. To test this hypothesis, we treated HS5 cells with 5 µM **CuZA** for 24 h followed by drug withdrawal and the addition of fresh medium supplemented with 0.5 mM cysteine. In this setting, the cells were pre-loaded with **CuZA** whereas the external copper content was negligible. Nevertheless, the addition of cysteine for 24 h yielded similarly high percentage of PI-positive cells (78 ± 11%) as if extracellular **CuZA** was present (82 ± 10%; mean ± standard errors, n = 3 measurements). Therefore, even limited amounts of intracellular **CuZA** were sufficient for triggering the reductive cytotoxicity. These observations strongly suggested that ROS generation upon electrochemical Cu^2+^-to-Cu^1+^ transition is a powerful mechanism of cell elimination.

**Figure 5:**
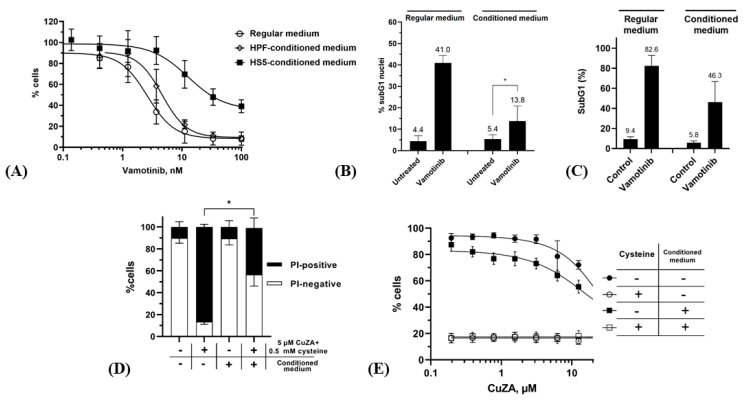
Combination of CuZA and cysteine overcomes prolonged K562 cell protection from vamotinib in the medium conditioned by bone marrow fibroblasts. (**A**–**C**) Protective effect of HS5-conditioned media on vamotinib cytotoxicity in K562 cells. (**A**) 72 h of exposure (**B**) 72 h with 12.5 nM vamotinib. (**C**) 72 h with 12.5 nM vamotinib in the regular or conditioned media followed by washing off the drug and incubation in the respective drug-free media for 9 days. (**D**,**E**) Potency of **CuZA** + cysteine against K562 cells in the regular and conditioned media. (**D**) % PI-positive K562 cells exposed to 5 µM CuZA and 0.5 mM cysteine in the regular and HS-5-conditioned media for 48 h. (**E**) Equal cytotoxicity of the combination in regular and HS5-conditioned media (72 h). Experiments were performed as n = 3 biological replicates, each sample tested in duplicate. See Section 4 for details. * *p* < 0.05 (ANOVA unpaired *t*-test).

We took advantage of this mechanism for induction of death in cells with altered response to specific chemotherapeutics. Indeed, CML cells that were initially sensitive to Bcr-Abl-targeting TKI can survive in the course of continuous treatment leading to a relapse [27,28]. In particular, the viability of CML cells that colonized the bone marrow can be promoted by stromal elements that form a survival niche for tumor cells. Therefore, the pro-apoptotic stimuli initiated by targeting Bcr-Abl with TKI are countered by signaling engaged by integrin-mediated adhesion of CML cells to extracellular matrix as well as by soluble factors such as cytokines [29,30,31]. We tested whether microenvironmental factors produced by HS5 bone marrow-derived fibroblasts can attenuate CML cell death in response to Bcr-Abl inhibition. The K562 CML cells cultured in the presence of HS5 fibroblasts or bone marrow mesenchymal stem cells were partially protected from the prototypic Bcr-Abl antagonist, imatinib mesylate [32,33,34]. Likewise, we observed a pro-survival effect of the bone marrow microenvironment on K562 cells treated with vamotinib (PF-114), a 3rd generation TKI [35,36,37]. In the regular medium, IC_50_ of this compound was 2.4 ± 1.0 nM after 72 h. However, this parameter could not be accurately calculated from the survival curve if cells were treated in HS5-conditioned medium: ~40% of cell population remained viable even at 100 nM vamotinib (Figure 5A). However, the medium conditioned by bone marrow-unrelated fibroblasts (HPF cell line) had no protective effect on the response of K562 cells to vamotinib, supporting the role of the organ-specific microenvironment. Lower percentages of subG1 events in K562 cells treated with vamotinib for 72 h confirmed the attenuated cytotoxicity in the conditioned medium (Figure 5B). Importantly, the protective effect of the conditioned medium was prolonged: by 12 days in the regular medium >80% cells displayed the fraction of fragmented DNA (Figure 5C) whereas this parameter was smaller in the conditioned medium. These data indicated that conditioning by HS5 fibroblasts conferred a long-term survival to vamotinib-treated K562 CML cells, substantiating a serious limitation of the efficacy of targeted therapy in the bone marrow microenvironment.

In striking contrast, no protection by the conditioned medium was detectable for combinations of submicromolar concentrations of **CuZA** and 0.5 mM cysteine. By 48 h ~40% cells were PI-positive (Figure 5D); however, the resazurin tests showed a dramatically decreased viability of total population of K562 cells (signals dropped down to the background levels of the assays both in the regular and conditioned media; Figure 5E) (see [38] for the approaches to determine the statistical differences between ‘cysteine’ vs. ‘no cysteine’ groups). Thus, the combination of **CuZA** and the physiological reducing agent (both at non-toxic concentrations) potently killed CML cells in the situation where the response to Bcr-Abl inactivation was altered. 

## 3. Discussion

Electrochemical reduction of Cu^2+^ to Cu^+^ in different chemical contexts is an efficient approach to kill cells. The multiplicity of death pathways triggered by ROS generated in this reaction allows to circumvent chemotherapeutic drug resistance. Most importantly, the early loss of the plasma membrane integrity, a key mechanism of cell vulnerability, remains operational in pleiotropically resistant cells. However, development of practical applications of Cu^2+^ → Cu^+^ redox transition mechanism in cancer treatment presumes a number of conditions. First, the organic milieu should be ‘permissive’ for the copper (II) cation to be available for electrochemical reduction. In other words, the reduction potential in the Cu–organic complex must be lower than the oxidation potential of the reducing agent [17]. Second, to minimize general toxicity, ROS generation should be localized to the site(s) of tumor cell accumulation. This prerequisite highlights the importance of organ delivery of Cu(II) cations using specific chemical scaffolds as vectors. However, biocompatibility of metal–organic complexes can be hampered by limited solubility in aqueous media. Furthermore, intracellular accumulation of copper–organic complexes should ensure an amount of copper sufficient for ROS burst upon metal reduction. Moreover, on their route to the target organ, Cu(II) cations can be redistributed from the initial complex to other carriers such as blood plasma proteins. Or else a portion of exogenous Cu(II) cations can undergo redox transition in vivo by physiological reducing compounds, e.g., glutathione. Together, these factors can interfere with the delivery of copper to the target organ.

In the present study, we report the initial evaluation of **CuZA** complexes designed as a tentative tool for metal delivery to the bone. Bisphosphonates (ZA in particular) have demonstrated a good clinical efficacy in prevention of bone resorption in metastatic lesions as well in non-malignant osteopathy. Thus, it is plausible to hypothesize that ZA could serve for copper transport to the bone tissue. In this scenario, **CuZA** emerges as a dual activity agent expected to act as a bone protector (due to the bisphosphonate moiety) and a depot of copper; the latter would be reduced not before the sufficient quantities of cysteine or ascorbate are added.

The one-step synthesis of **CuZA** from three salts of divalent copper (i.e., perchlorate, sulfate and chloride) yielded identical complexes in which three Cu^2+^ cations coordinated two ZA molecules. In electrochemical experiments, we replicated the effect of Cu^2+^ → Cu^+^ redox transition in **CuZA** in the presence of NAC previously demonstrated for CuO and many (but not any) Cu(II)-containing compounds contingent on the organic scaffold [17]. Therefore, the bisphosphonate milieu did not limit the reductive potential of Cu^2+^ cations, making **CuZA** promising for biological investigation. However, **CuZA** was virtually insoluble in polar and non-polar media. Nevertheless, we obtained water-soluble formulations by varying the ratios of aqueous solutions of ethanol and NaOH followed by stabilization with Kollidon 17PF. These preparations were tested for the ability to trigger oxidative damage in cultured cells.

Along with these findings, we observed that intracellular accumulation of water-soluble **CuZA** was relatively low as determined by atomic force spectrometry-assisted measurements of copper content. The majority of copper remained in the extracellular medium. Still, the quantities of intracellular copper after removal of the medium were enough for reductive cytotoxicity. These results further proved the anticancer potential of this approach even if limited amounts of copper are delivered to the tumor site.

The bone marrow microenvironment represents a protective niche for resident tumor cells [39,40,41]. A complex network of pro-survival signaling includes interactions of tumor cells with extracellular matrix and non-malignant stromal elements. These direct contacts, along with distant regulatory mechanisms mediated by soluble factors (chemokines), provide an opportunity for tumor cells to escape the cytotoxicity of chemotherapeutics. Bcr-Abl inhibitors have been shown to be less efficacious against CML cells in the bone marrow than in the peripheral blood [3,4,5]. The protective effect is not limited to CML: the acute myeloid leukemia cells are rescued from chemotherapeutic drugs in the hematopoietic niche [42]. Whatever the mechanism of survival in the bone microenvironment, the resident leukemia cells exhibit a pleiotropic drug resistance acquired in the course of repetitive rounds of treatment. Targeted therapy is no longer efficient; new approaches are needed to cope with the disease progression. One critical prerequisite is the induction of non-apoptotic death pathways.

Our model of altered response of CML cells to the Bcr-Abl antagonist presumed the use of the culture medium conditioned by bone marrow-derived fibroblasts. This medium partially rescued K562 cells from vamotinib, a 3rd generation Bcr-Abl inhibitor that recently entered clinical trials [31]. Molecular mechanisms that confer epigenetic resistance of K562 cells to Bcr-Abl inhibition in the conditioned medium are under investigation by our group. Importantly, in the HS5-conditioned medium, as well as in the regular medium, the combinations of **CuZA** and cysteine (each component alone at non-toxic concentrations) were cytocidal. These results were in line with the reported exceptional potency of individual copper-containing compounds upon electrochemical metal reduction [8].

This study provides evidence in favor of Cu(II) reductive cytotoxicity as an alternative to kill cells that survived certain therapeutic stimuli. A major prerequisite for an approach aimed at circumventing pleiotropic resistance is the ability to trigger multiple death mechanisms [43]. Most importantly, cell death upon Cu^2+^-to-Cu^+^ redox transition involves the damage of the plasma membrane and membrane organelles ([8], this study). The necrotic pathway remains functional in cells otherwise irresponsive to a variety of apoptotic stimuli. Therefore, necrosis can emerge as a method of choice in advanced disease. Moreover, the selective targeting of CML cells in the organ can be insufficient due to pro-oncogenic influence of the microenvironment. Strategies of sparing non-malignant counterparts within the tumor site may be palliative; we argue in favor of an indiscriminate elimination of tumor cells and the surrounding milieu as a salvation therapy in late stages of the disease. Definitely, the oxidative ablation of the bone marrow demands an exceptionally thorough patient care to prevent unfavorable systemic complications, primarily infection.

### Study Limitation

Advantages of the reported approach are the readiness of the thrifty synthesis, the stability of water-soluble **CuZA** formulations, and their cytocidal potency in combinations with cysteine in electrochemical assays and in cell culture. Although the principle is proved, the efficacy of **CuZA**–cysteine combinations in vivo remains to be investigated in detail. Among the critical issues is the ability of **CuZA** complexes to keep the metal protected from the attack by reducing agents in the serum and tissues, aiming at the delivery of maximal amounts of Cu^2+^ to the organ. Next, the development of the drug candidate presumes the formation of the bone depot via maximum ZA-assisted Cu^2+^ delivery. ZA is expected to ensure the prolonged retainment of the metal in the bone; however, there is a possibility for Cu^2+^ exchange between **CuZA** and small molecular weight compounds and/or proteins in the site. Still, one may anticipate that such a re-complexation, if it takes place, should not attenuate the cytocidal potency of the local ROS burst generated upon Cu^2+^-to-Cu^1+^ transition inside the cells as well as in the extracellular milieu.

## 4. Materials and Methods

### 4.1. General

All chemicals purchased from Merck (Darmstadt, Germany), Lancaster (Morecambe, UK) and ABCR (Karlsruhe, Germany) were reagent grade and used without purification. Melting points were determined using an OptiMelt MPA100—Automated melting point system (Stanford Research Systems (SRS), Sunnyvale, CA, USA), 1 °C/min, 0.1 °C resolution. Infrared spectra were recorded on a Thermo Nicolet iS5 FTIR (Thermo Fisher Scientific, Waltham, MA, USA), number of scans 32, resolution 4 cm^−1^, sampling ATR. Elemental analysis of CHNS/O was performed using a Perkin Elmer Model 2400 Series II (Perkin Elmer, Waltham, MA, USA). Electronic spectra in the UV and visible regions were recorded on a Hitachi U-2900 instrument (Tokyo, Japan) with an operating wavelength range of 190–1100 nm in a quartz cuvette manufactured by Agilent Technologies with an optical path of 10 mm. For the ICP-MS study, an 8.0 mg sample of CuZA was dissolved in concentrated nitric acid, diluted, and the copper content was determined using the total internal reflection X-ray phase analysis; quantitative calculations were performed using the standard addition method. ESI mass spectra were recorded on an Agilent 6470 mass spectrometer (Agilent Technologies, Inc., Santa Clara, CA, USA) equipped with a triple quadrupole mass analyzer in positive ion detection mode. The following ion source parameters were used: nebulizer gas flow (nitrogen) 10 L min^−1^, desiccant gas flow (nitrogen) 11 L min^−1^, interface capillary voltage of 4.5 kV, interface temperature of 300 °C, and desolvation capillary temperature of 325 °C. MALDI mass spectra were obtained on a Bruker Autofl Ex Speed mass spectrometer (Bruker Daltonics Inc., Bremen, Germany), equipped with a solid-state UV laser (λ 355 nm) and a reflector, in positive ion mode at the lowest possible laser energy. An MTP 384 ground steel target (Bruker Daltonics Inc., Bremen, Germany) was used to record the MALDI mass spectra. Data collection and processing were performed using the Compass 1.4 software package (Bruker Daltonics Inc., Bremen, Germany).

### 4.2. Synthesis

Coordination compounds [Cu_3_(ZL)_2_(H_2_O)_6_] xH_2_O were prepared by sedimentation using ZA and copper salts (CuCl_2_ 2H_2_O, CuSO_4_ 5H_2_O, Cu(ClO_4_)_2_ 6H_2_O).

(1) ZA (82 mg; 0.3 mmol) was dissolved in 10 mL methanol and heated up to 64°C with constant stirring, then a solution of CuCl_2_ 2H_2_O (102 mg; 0.6 mmol) in 5 mL methanol was added and the mixture was stirred for 24 h at 64°C. The resulting blue-green precipitate was filtered off, washed with ethanol, water and air-dried. Yield 67%. The composition of the complex was determined by elemental analysis: Cu_3_(ZL)_2_(H_2_O)_6_. Elemental analysis: calcd for C_10_H_26_Cu_3_N_4_O_20_P_4_ 5H_2_O: C% 14.03; H% 4.17; N% 5.95. Found: C% 13.70, H% 4.09, N% 5.88. ICP-MS: 22.28% Cu was found (22.84% calculated); phosphorus and minor chloride impurities were also identified in the sample spectrum. IR spectra (KBr, cm^−1^): 3444, 3167, 1627, 1400, 1279, 1151, 1077, 1031, 990, 965, 842, 638, 603.

(2) ZA (82 mg; 0.3 mmol) was dissolved in 10 mL methanol and heated up to 64°C with constant stirring, then a solution of CuSO_4_ 5H_2_O (154 mg; 0.6 mmol) in 5 mL methanol was added and the mixture was stirred for 24 h at 64°C. The resulting blue-green precipitate was filtered off, washed with ethanol, water and air-dried. Yield 73%. The composition of the complex was determined by elemental analysis: Cu_3_(ZL)_2_(H_2_O)_6_. Elemental analysis: calcd for C_10_H_26_Cu_3_N_4_O_20_P_4_ 2H_2_O: C% 13.76; H% 3.46; N% 6.42. Found: C% 13.62, H% 3.19, N% 6.28. IR spectra (KBr, cm^−1^): 3444, 3167, 1627, 1400, 1279, 1151, 1077, 1031, 990, 965, 842, 638, 603.

(3) ZA (82 mg; 0.3 mmol) was dissolved in 10 mL methanol and heated up to 64°C with constant stirring, then a solution of Cu(ClO_4_)_2_ 6H_2_O (222 mg, 0.6 mmol) in 5 mL methanol was added and the mixture was stirred for 24 h at 64°C. The resulting blue-green precipitate was filtered off, washed with ethanol, water and air-dried. Yield 58%. The composition of the complex was determined by elemental analysis: Cu_3_(ZL)_2_(H_2_O)_6_. Elemental analysis: calcd for C_10_H_26_Cu_3_N_4_O_20_P_4_ H_2_O: C% 14.05; H% 3.30; N% 6.55. Found: C% 13.99, H% 3.02, N% 6.41. IR spectra (KBr, cm^−1^): 3444, 3167, 1627, 1400, 1279, 1151, 1077, 1031, 990, 965, 842, 638, 603.

### 4.3. Electrochemistry

An IPC Pro M potentiostat (IPC Pro M-Volta, Saint Petersburg, Russia) was used for electrochemical studies. The working electrode was a glassy carbon disk (d = 2 mm), the reference electrode was Ag/AgCl/KCl (sat.). The auxiliary electrode was a platinum plate, and the supporting electrolyte was 0.1 M Bu_4_NClO_4_ solution in DMSO. Potential scan rates were 100 mV/s and 20 mV/s^−1^ in CV and RDE methods, respectively. Measurements were carried out in a dry argon atmosphere; samples were dissolved in a de-aerated solvent.

### 4.4. Preparation of Water-Soluble Formulations

The required amounts of powdered **CuZA** complex and Kollidon 17PF (BASF, Ludwigshafen, Germany) were placed in a glass beaker and dissolved in the respective volume of 95% ethanol with stirring (400 rpm) and heating (60 °C) on a magnetic device for 20 min. To a clear blue solution, 0.1 N NaOH was added dropwise with stirring at 200 rpm to produce a light blue solution. The latter was filtered through 0.45 µM filter and stored at 4 °C for at least 6 mo. without opacity. Preparations were used as 10 mM stock solutions in the experiments.

### 4.5. Measurements of Copper in Cell Lysates and Extracellular Medium

The copper content was a measure of intracellular and extracellular **CuZA** accumulation. Cells were treated with 5 µM **CuZA** in the culture medium for 3–24 h. The medium containing extracellular **CuZA** was collected. Monolayers were washed with saline and lysed in dH_2_O. Lysates were centrifuged (10,000× *g* 3 min) to pellet the water-insoluble material. Supernatants containing cell-associated **CuZA** were processed for quantitative measurement of copper in the extracellular medium and in lysate supernatants on a quadrupole inductively coupled mass spectrometer (ICP-MS) PlasmaQuant MS Elit (Analytik Jena AG, Jena, Germany) after 125–145-fold dilution in 3% (*v*/*v*) HNO_3_. Each sample was analyzed in triplicate. Data were acquired and processed with the ASpect MS software package (version 4.3, Analytik Jena, Jena, Germany). Indium (1 µg/L) was an internal standard. ^115^In, ^63^Cu and ^65^Cu isotopes were determined using a collision cell (gaseous He, 40 mL/min) and without collision cell. For calibration, the multi-element Standard E solution (ICP-MS-E-100, High-Purity Standards) was diluted to reach final concentrations 0.2, 0.5, 1, 2, 5, 10 and 20 µg/L.

### 4.6. Cell Culture and Cytotoxicity Assays

Human HS5 bone marrow fibroblasts, K562 CML cells (American Type Culture Collection; Manassas, VA, USA) and HPF foreskin fibroblasts (gift of Dr. S.M.; Dashinimaev, Engelhardt Institute of Molecular Biology, Moscow, Russia) were cultured in RPMI-1640 supplemented with 10% fetal bovine serum (HyClone; Logan, UT, USA) and 50 μg/mL gentamicin at 37 °C, 5% CO_2_ in a humidified atmosphere. Water-soluble formulations of **CuZA** were serially diluted in the culture medium from 10 mM stock solution immediately before the experiments. Metal-free ZA was dissolved in DMSO (10 mM). *L*-cysteine was dissolved in culture medium as 50 mM stock solution, then pH was adjusted to 7.2–7.4 with 1 M NaOH. Viability of HS5 cells treated with **CuZA** alone or in combination with cysteine was determined in MTT tests [8]. Vamotinib (gift of Dr. G. Chilov; Valenta Pharm, Russia) was dissolved in DMSO as 10 mM stock solution. The K562 cells were exposed to the increasing concentrations of vamotinib (freshly reconstituted from the stock solution in the culture medium) for 72 h. In parallel, cells were treated with vamotinib for 72 h in the medium pre-conditioned by HS5 or HPF (bone marrow-unrelated cell type control) fibroblasts for 48 h. To assess the prolonged survival, K562 cells were treated with vamotinib in the regular or HS5-conditioned medium for 72 h, then resuspended in respective drug-free media and further incubated for another 9 days, changing the media every 3 days (total 12 days). For drug combination experiments, K562 cells were treated with 5 µM **CuZA** and 0.5 mM cysteine in the regular or HS5-conditioned medium for 48 h. Cell viability was assessed in resazurin tests [44] as well as by flow cytometry. Sample processing and evaluation of subG1 events (in lysed cells) and PI-positive whole cells (no lysis) have been described by us [8]. Measurements were performed on a CytoFlex flow cytometer (Beckman Coulter Inc., Brea, CA, USA). Twenty thousand fluorescent events were collected per sample (n = 3 biological replicates, each measured in duplicate). Data was analyzed using CytExpert 2.3 software (Beckman Coulter, Brea, CA, USA).

### 4.7. Statistics

One-way or two-way analyses of variance (ANOVA) followed by Sidak’s post hoc test for multiple comparisons were used (GraphPad Prism 9; GraphPad Software, San Diego, CA, USA). The *p* value < 0.05 was taken as evidence of statistical significance.

## 5. Conclusions

Exploring the efficacy of the oxygen burst upon reduction of copper (II) in the context of organic compounds, we performed a one-step synthesis of complexes containing two molecules of the bone-affine drug ZA coordinated by three Cu^2+^ cations (**CuZA**). Electrochemical techniques confirmed Cu^2+^ → Cu^1+^ transition in the presence of the reducing agent. Poor water solubility of the initial **CuZA** preparations was overcome by obtaining stable aqueous formulations suitable for cell culture. These formulations together with cysteine (each component at non-toxic concentrations) triggered death in K562 CML cells in regular medium. Importantly, these combinations were similarly cytocidal against K562 cells in the medium conditioned by HS5 bone marrow-derived fibroblasts where the efficacy of the 3d generation Bcr-Abl inhibitor vamotinib was limited. Thus, the new **CuZA** complexes serve as a tool for preclinical investigation of dual potency, bone-directed antitumor drug candidates.

## Figures and Tables

**Table 1:** Extracellular and intracellular copper content (µg/L) determined by ICP-MS.

Time, h	HS5 Cells		K562 Cells	
	Medium	Cell Lysate	Medium	Cell Lysate
0	14 ± 4	25 ± 6	12 ± 2	15 ±5
3	837 ± 16	130 ± 22 *	956 ± 24	111 ± 13 *
8	988 ± 10	106 ± 10 *	924 ± 16	116 ± 12 *
24	984 ± 25	220 ± 28 *	701 ± 30	322 ± 34 *

Cells were treated with 5 µM **CuZA** for indicated time intervals. Media were collected; cell monolayers were washed with saline and lysed in dH_2_O. Samples were processed for measurements of copper content (see Section 4). Values are mean ± confidence interval (n = 3 replicates, each sample measured in triplicate). * *p* < 0.05 compared with the respective Medium group.

## Data Availability

The original contributions presented in this study are included in the article/Supplementary Material. Further inquiries can be directed to the corresponding author.

## Supplementary Materials

The following supporting information can be downloaded at: https://www.mdpi.com/article/10.3390/ijms27062800/s1.
