# Supplementary material for: The Complex of Copper (II) and Zoledronic Acid: Relevance to Oxidative Death of Leukemia Cells in the Bone Marrow
Source: Int J Mol Sci. 2026 Mar 19;27(6):2800. doi: 10.3390/ijms27062800 (PMC13026577; doi:10.3390/ijms27062800)
Supplement: Supplementary file 1 [file ijms-27-02800-s001.zip › ijms-4118829-supplementary.pdf]

Electronic Supplementary Materials

The Complex of Copper(II) and Zoledronic Acid: Relevance to Oxidative Death of Leukemia Cells in the Bone Marrow

Contents

Spectral data of CuZA compound

Figure S1. IR spectrum of

Figure S2. Electronic spectrum of CuZA in UV and visible regions

Figure S3. ESI-MS spectrum

Figure S4. ESI-MS spectrum in the region 750-1400 m/z

Figure S5. MALDI spectrum on the 1,8,9-anthracentriol (AT) matrix

Figure S6. MALDI spectrum on α-cyano-4-hydroxycinnamic acid (CHCA) matrix

Figure S7. MALDI spectrum on the *trans*-2-[3-(4-*tert*-butylphenyl)-2-methyl-2-propenylidene]malononitrile (DCTB) matrix

Figure S8. MALDI spectrum on the rubrene matrix

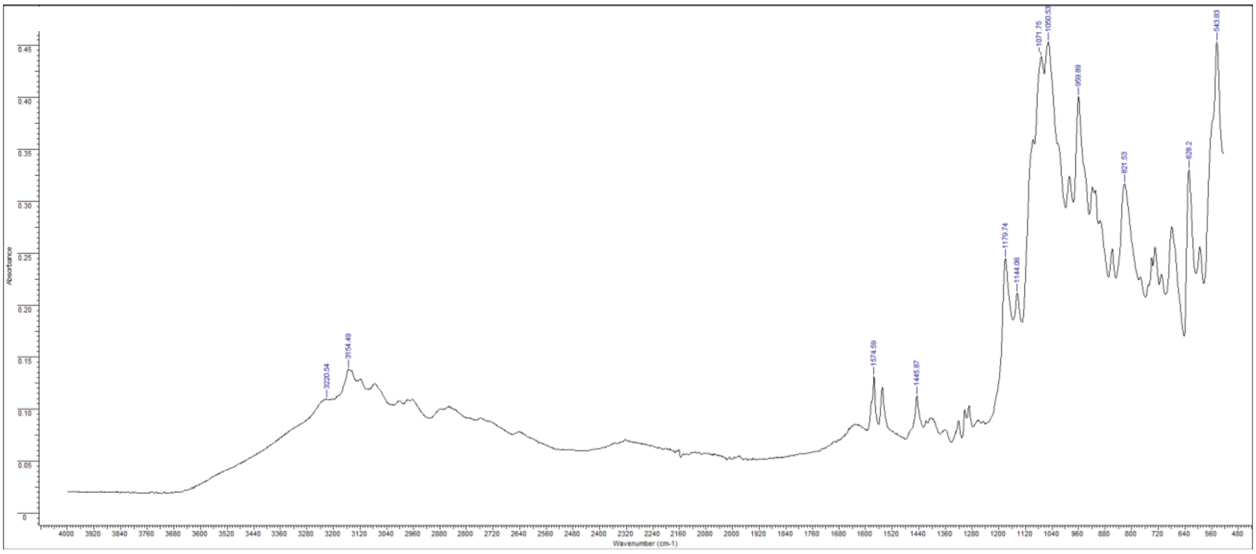

Figure S1. IR spectrum of **CuZA**.

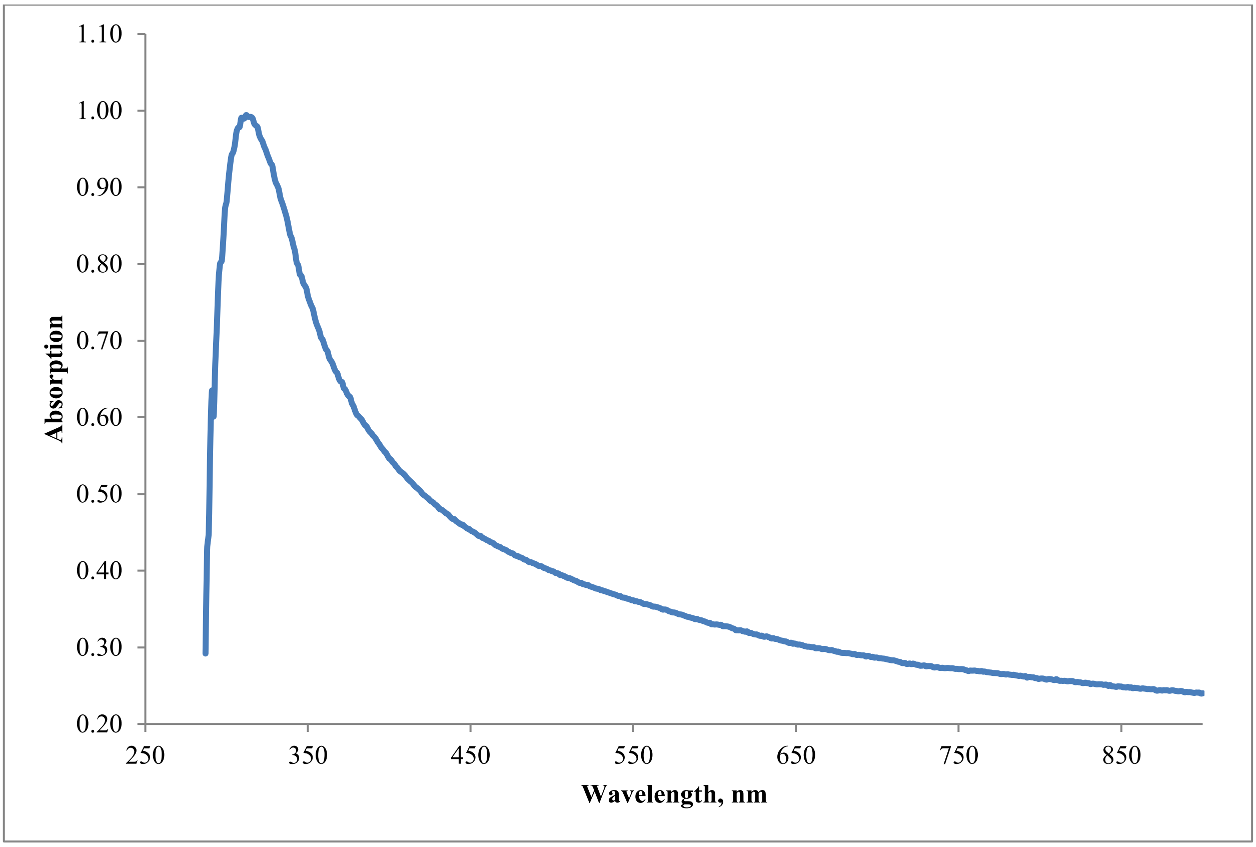

Figure S2. Electronic spectrum of **CuZA** in UV and visible regions.

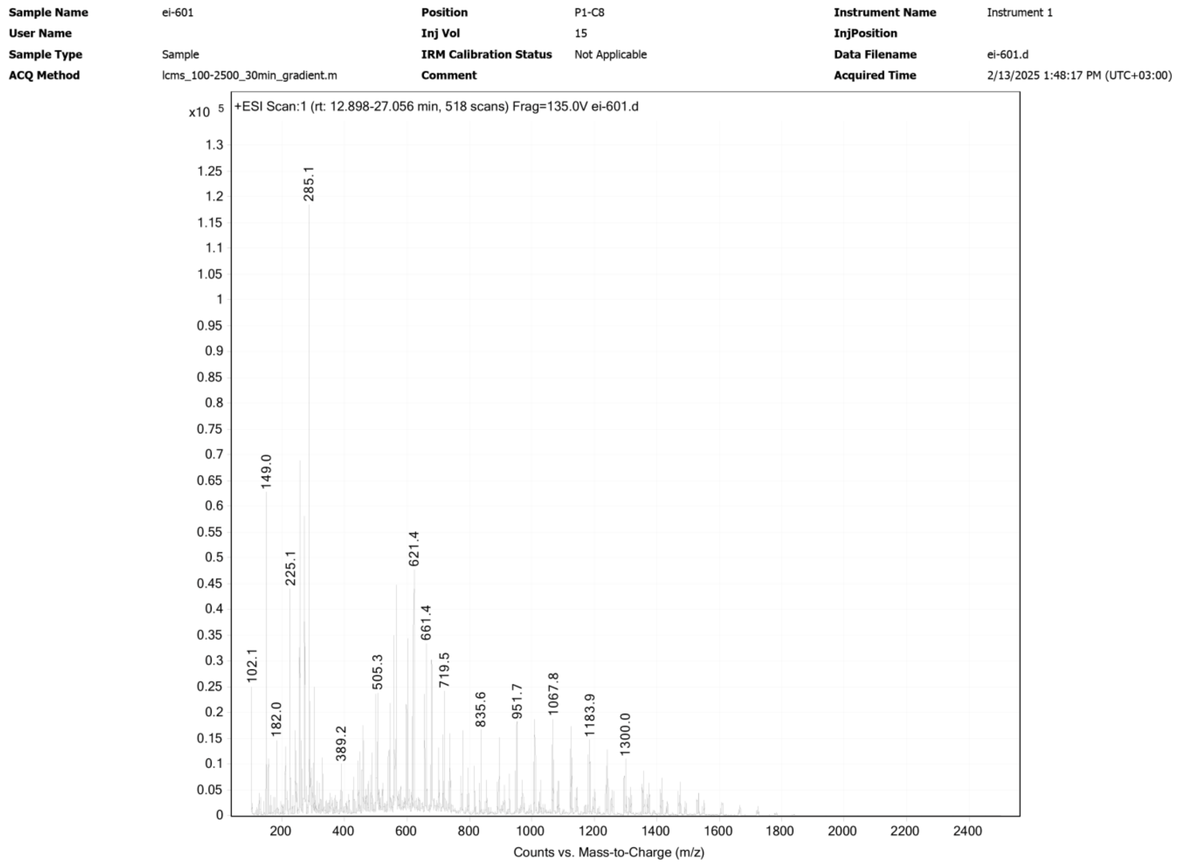

Figure S3. ESI-MS spectrum of **CuZA**.

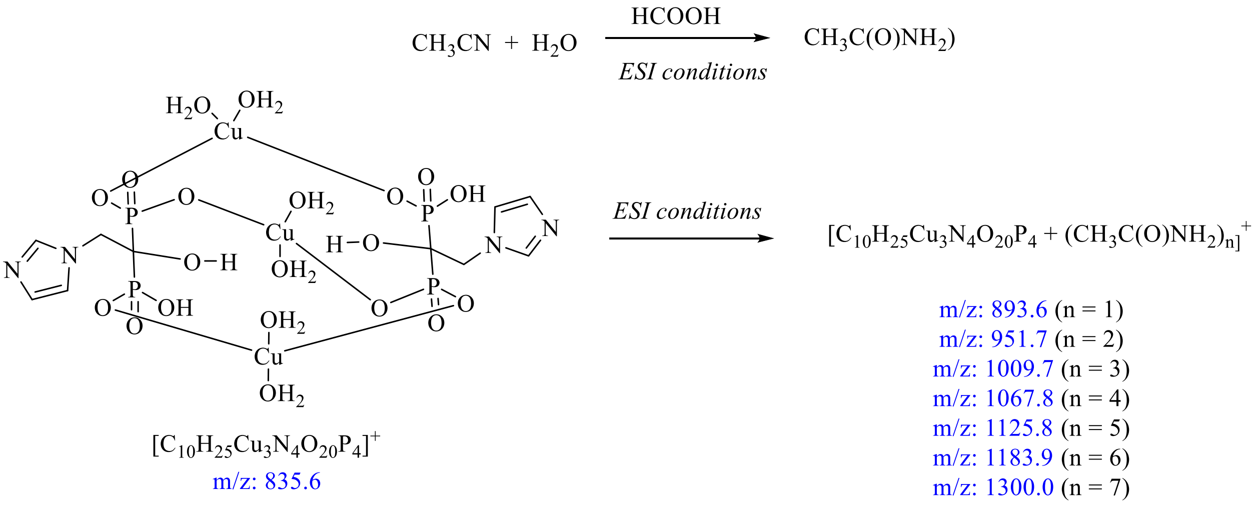

Figure S4. Proposed scheme of the formation of additional ions (ESI conditions).

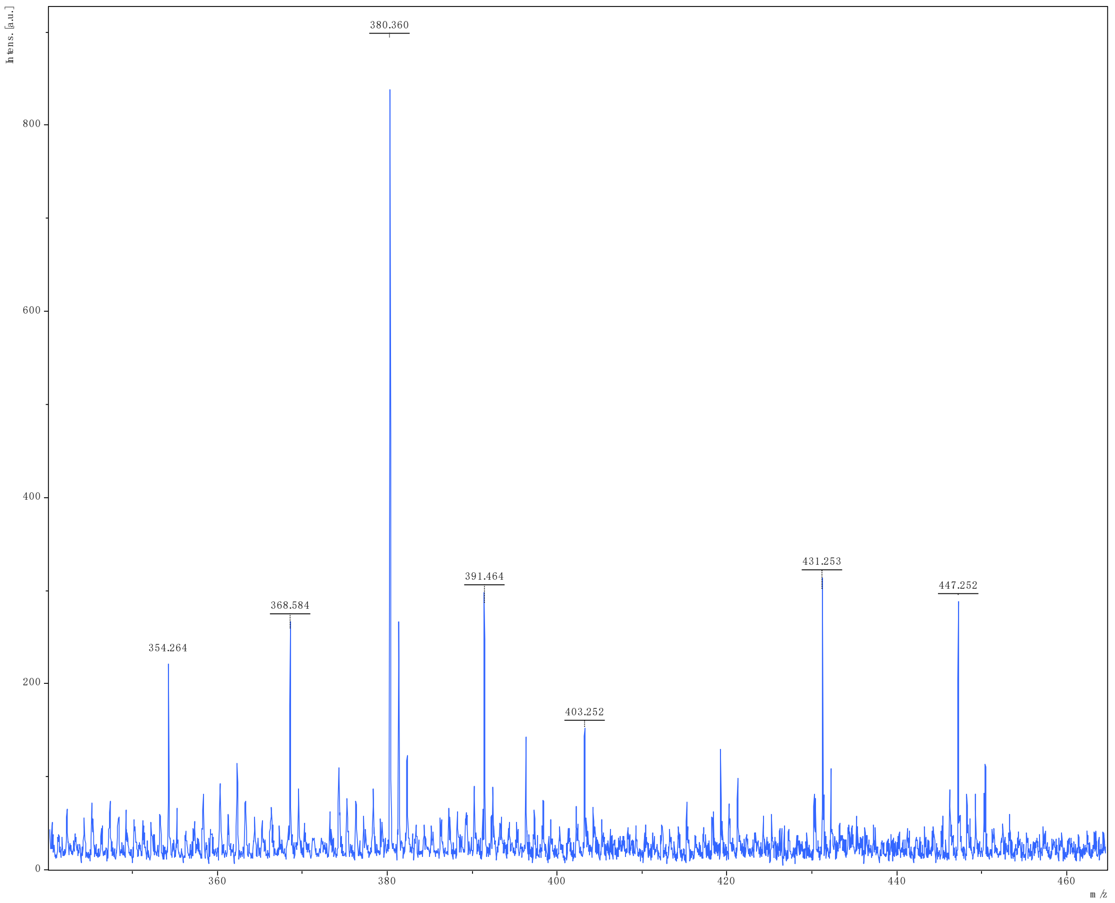

Figure S5. MALDI spectrum of **CuZA** on 1,8,9-anthracentriol (AT) matrix.

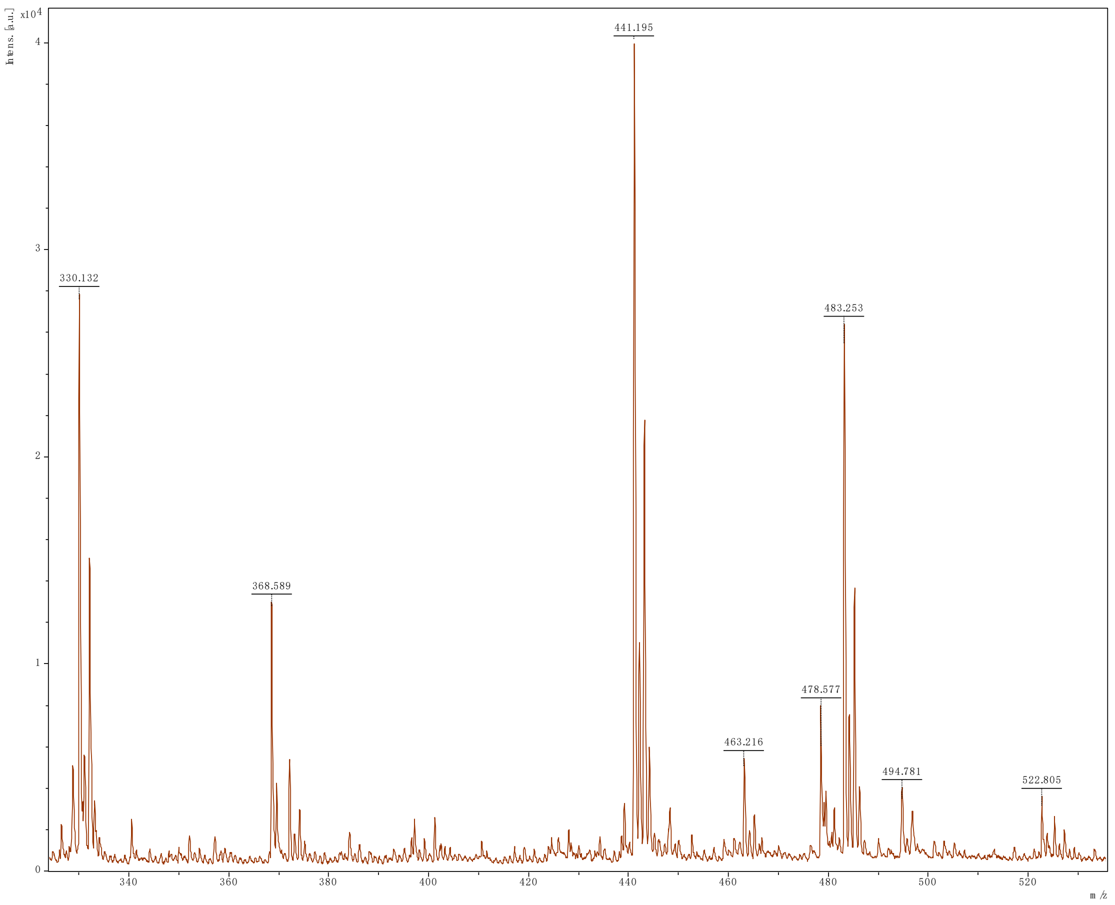

Figure S6. MALDI spectrum of **CuZA** on α-cyano-4-hydroxycinnamic acid (CHCA) matrix.

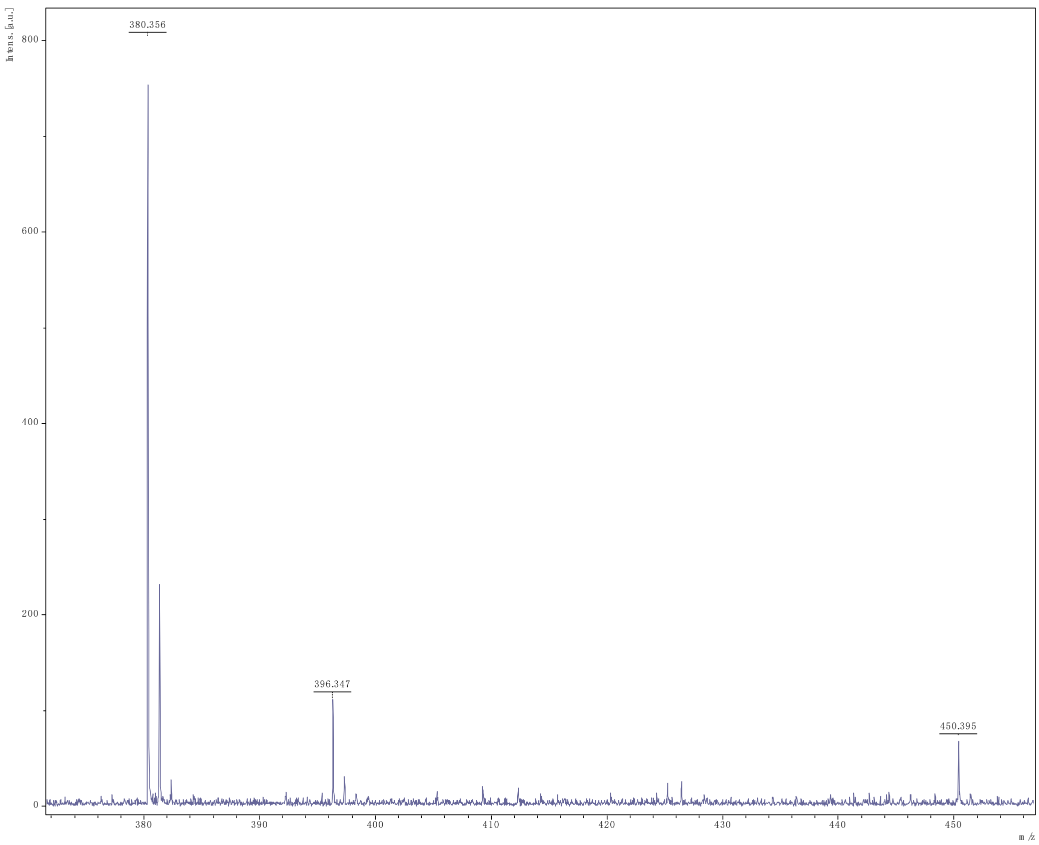

Figure S7. MALDI spectrum of **CuZA** on trans-2-[3-(4-tert-butylphenyl)-2-methyl-2-propenylidene]malononitrile (DCTB) matrix.

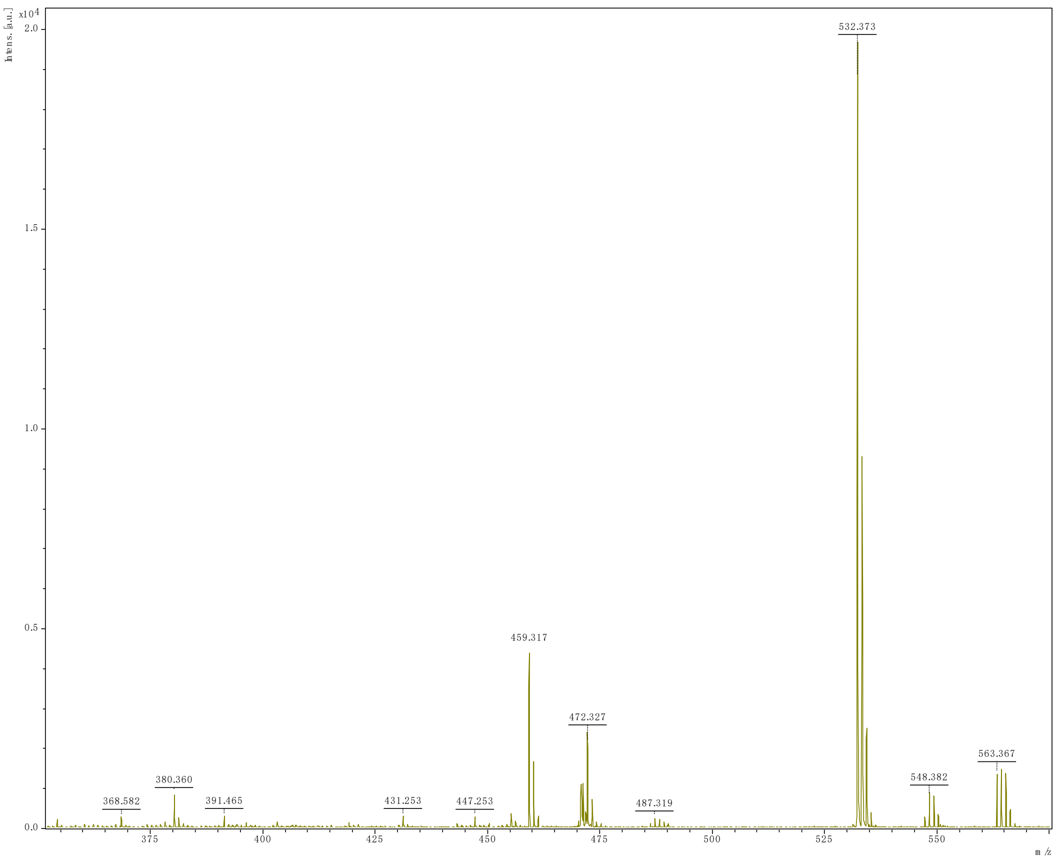

Figure S8. MALDI spectrum of **CuZA** on rubrene matrix.
